# Medical and Surgical Management of Hidradenitis Suppurativa

**DOI:** 10.3390/jcm15135238

**Published:** 2026-07-04

**Authors:** John W. Frew, Falk G. Bechara

**Affiliations:** 1 The Skin Hospital, 121 Crown St Darlinghurst, Sydney, NSW 2010, Australia; 2 School of Clinical Medicine, University of New South Wales, Sydney, NSW 2060, Australia; 3 ICH—International Center for Hidradenitis Suppurativa/Acne Inversa, Department of Dermatology, Venereology and Allergology, Ruhr-University Bochum, 44801 Bochum, Germany

**Keywords:** hidradenitis suppurativa, acne inversa, surgery, biologics, adalimumab, secukinumab, bimekizumab, wide excision, deroofing, guideline review

## Abstract

**Background:** HS is a chronic inflammatory skin disease in which inflammatory nodules and abscesses coexist with tunnels, fibrosis, and scarring. This dual biology explains why medical therapy often improves inflammatory dissease activity without fully addressing fixed tissue damage, whereas surgery can achieve durable local control but does not treat diffuse inflammatory burden. Contemporary international guidelines increasingly endorse multimodal and medicosurgical care. **Objective:** To critically compare the evidence supporting medical and surgical management of HS, with emphasis on outcomes, indications, limitations, and clinical decision-making relevant to contemporary practice. **Methods:** A structured review was undertaken using PubMed/MEDLINE, the Cochrane Library, and major dermatology guideline sources, with searches updated to 7 May 2026. Priority was given to clinical guidelines, systematic reviews and meta-analyses, randomized controlled trials, and higher-quality observational studies. Evidence was synthesized narratively because endpoints, populations, and follow-up intervals differed markedly across medical and surgical studies. **Results:** Medical evidence is strongest for biologic therapy in moderate-to-severe inflammatory HS. Weekly adalimumab improved week-12 HiSCR in the phase 3 PIONEER trials; secukinumab improved week-16 and week-52 outcomes in SUNSHINE/SUNRISE; and bimekizumab improved week-16 HiSCR50 in BE HEARD I/II. Surgical evidence is strongest for wide excision in structurally advanced disease, particularly when compared with local excision or incision and drainage. Meta-analytic data consistently show lower recurrence after wide excision than after local excision, and lower recurrence after flap or graft reconstruction than after primary closure. Combined therapy is increasingly supported: peri-operative adalimumab improved outcomes in SHARPS, and surgery plus adalimumab outperformed adalimumab alone in a pragmatic 12-month RCT. **Conclusions:** HS is best managed by matching treatment to disease phenotype. Medical therapy is essential for inflammatory control; surgery is essential for persistent tunnels, fibrosis, and scarred regional disease. The strongest overall clinical position is an integrated, multidisciplinary model in which systemic therapy reduces inflammatory load and surgery definitively treats irreversible tissue damage.

## 1. Introduction

HS presents as painful recurrent nodules, abscesses, draining tunnels, and scarring in intertriginous sites, with a major physical and psychosocial burden. A central therapeutic difficulty is that the disease contains both inflammatory lesions that can regress with medical therapy (e.g., nodules and abscesses) and chronic structural lesions that are less reversible once fibrosis and tunnel formation are established (draining tunnels). That pathobiological distinction now underpins most modern treatment algorithms [1,2,3,4,5,6,7,8].

Older guidance often placed surgery late in the pathway, after repeated medical failure. More recent guidance is less linear and more phenotype-driven. The French guideline explicitly recommends a medicosurgical approach for all patients, the Australasian guideline discourages sequential monotherapy and promotes multimodal care, and the European S2k guideline distinguishes inflammatory HS from predominantly non-inflammatory disease in deciding between medication-led and surgery-led strategies. Comparative guideline review further shows broad international agreement that topical therapy and tetracyclines suit milder disease, adalimumab has been the historic first-line biologic, and deroofing or wide local excision should be used for recurrent fixed lesions and advanced regional disease [4,5,7,8]. Importantly, modern guidelines increasingly view procedural interventions as complementary treatment modalities rather than as markers of therapeutic failure, supporting early deroofing or local excision for recurrent fixed lesions even when overall inflammatory burden remains relatively limited.

The practical question for clinicians is therefore not “medicine or surgery?”, but rather which component of disease is dominant, how rapidly inflammatory control can be achieved, and when local destruction has become sufficiently fixed to justify procedural or excisional intervention. This review addresses that question by comparing the best available evidence for medical management, surgical management, and combined medical-surgical care.

## 2. Methods

This manuscript was prepared as a structured narrative review rather than a registered systematic review. PubMed/MEDLINE, the Cochrane Library, and journal or society sources for dermatology guidelines were searched up to 7 May 2026 using combinations of the terms *hidradenitis suppurativa*, *acne inversa*, *medical management*, *surgery*, *wide excision*, *deroofing*, *adalimumab*, *secukinumab*, *bimekizumab*, *guideline*, *systematic review*, and *meta-analysis*. No language restrictions or date restrictions were put in place for the search strategy.

Eligibility prioritized four evidence layers: first, international or national clinical guidelines; second, systematic reviews and meta-analyses; third, randomized controlled trials; and fourth, observational studies reporting clinically interpretable outcomes such as HiSCR, IHS4, DLQI, recurrence, healing time, or postoperative complications. Case reports and highly selective small case series were generally excluded from the main synthesis unless primarily cited by guidelines. Any overlapping cohorts present in more than one meta-analysis were included in only the larger of the overlapping analyses. Because surgical studies tend to report local recurrence and wound outcomes, whereas medical trials usually report short-term inflammatory response, the evidence was synthesized narratively rather than pooled de novo.

## 3. Results

The medical evidence base for HS is strongest in moderate-to-severe inflammatory disease. The historic pivot was the PIONEER program, where weekly adalimumab improved week-12 HiSCR over placebo in both phase 3 trials: 41.8% versus 26.0% in PIONEER I and 58.9% versus 27.6% in PIONEER II. These trials established that biologic therapy can meaningfully reduce inflammatory lesion burden, although a substantial proportion of patients still failed to reach response criteria [9,10]. Importantly, participants enrolled in the pivotal biologic trials generally had moderate-to-severe disease with substantial baseline inflammatory burden, including multiple abscesses and inflammatory nodules, and frequent prior exposure to systemic therapies. Consequently, even the reported HiSCR50 rates of approximately 42–59% imply that 40–60% of patients remained classified as non-responders at primary endpoints, highlighting a persistent unmet need and supporting interest in multimodal strategies that combine inflammatory control with procedural management of structural disease.

The subsequent phase 3 biologic literature broadened but did not fundamentally alter that message: biologics are effective for inflammatory control, but they do not directly remove established tunnels or scarred tissue. In SUNSHINE and SUNRISE, secukinumab every 2 weeks met the primary endpoint in both trials, yielding week-16 HiSCR rates of 45% versus 34% in SUNSHINE and 42% versus 31% in SUNRISE, with sustained benefit to week 52; the program enrolled 1084 patients across 40 countries. BE HEARD I/II extended comparable phase 3 evidence to bimekizumab, where week-16 HiSCR50 in pivotal arms was broadly 48–54% compared with 29–32% for placebo. Taken together, these trials support earlier biologic escalation for moderate-to-severe inflammatory HS, especially when the disease is diffuse or associated with major quality-of-life impairment [11,12].

Surgical evidence answers a different question: not whether inflammation decreases over 12–16 weeks, but whether a diseased anatomical field can be rendered recurrence-free or substantially quieter over months to years. In the 2021 systematic review and meta-analysis by Riddle et al. [13], wide excision had the lowest pooled recurrence among common excisional strategies at 8% (95% CI 2–16%), whereas local excision had 34% recurrence. Within wide/radical excision, flap repair had the lowest pooled recurrence, while delayed primary closure had the highest. The authors concluded that wider excision and flap-based reconstruction were associated with lower postsurgical recurrence, albeit with important heterogeneity and methodological limitations [13].

Other surgical syntheses broadly converge with that signal. Tang et al. estimated an overall postoperative complication rate of 11.1% and a pooled recurrence of 16.2%, underscoring that surgery is effective but not trivial. Ovadja and colleagues found lower recurrence when excision aimed for radical margins; in a separate meta-analysis of reconstruction after wide excision, primary closure carried the highest recurrence, while flap and graft techniques performed better. The recent Cucu network meta-analysis refined this further, showing lower recurrence with local/distant flaps (OR 0.450, 95% CI 0.260–0.780) and split-thickness grafting (OR 0.549, 95% CI 0.334–0.903) compared with primary closure [14,15,16,17].

Important nuance comes from modern recurrence-pattern work. In the retrospective study by Deckers et al. [17], wide excision with secondary intention healing across 253 procedures led to recurrence in 37.6% during a mean follow-up of 36.2 months, yet total remission of the operated anatomical area was still achieved in 49% of procedures, and patient satisfaction remained high. More recent studies suggest this apparent heterogeneity largely reflects inconsistent definitions of recurrence. Many studies report any subsequent inflammatory activity within an operated region as recurrence, whereas others distinguish true tunnel recurrence (reflecting failure to eradicate structurally diseased tissue) from inflammatory nodules or abscesses arising adjacent to the original surgical field. This distinction is clinically important because tunnel recurrence may represent surgical failure, whereas inflammatory relapse may instead reflect ongoing systemic disease activity. Cuenca-Barrales et al. [18] and García-Moronta et al. [19] argued that tunnel recurrence may better represent surgical failure than the reappearance of superficial abscesses or inflammatory nodules alone. In the 2026 cohort of 206 procedures, overall recurrence was 18.5%, with tunnel recurrence 8.3% and abscess/inflammatory nodule recurrence 10.2%; larger excised area independently increased overall recurrence risk, while obesity, postoperative inflammatory burden, and the extent of disease also mattered.

The most clinically relevant comparison studies are those evaluating combined care. In SHARPS, adalimumab plus surgery improved week-12 all-body HiSCR to 48% versus 34% with placebo plus surgery, with a treatment difference of 14% (95% CI 0–27%) and no new safety concerns, supporting continuation of adalimumab through the peri-operative period in appropriately selected patients. In a later pragmatic RCT, adalimumab plus surgery versus adalimumab alone produced a significantly larger 12-month IHS4 reduction (−19.1 ± 11.3 vs. −7.8 ± 11.8; *p* < 0.001) and greater DLQI improvement (−8.2 ± 6.2 vs. −4.0 ± 7.7; *p* = 0.02) [20,21].

The prospective cohort by Salvador-Rodríguez et al. [22] adds a useful peri-operative nuance. Compared with surgery alone, surgery plus neoadjuvant biologic therapy showed numerically lower 24-week recurrence (9.52% vs. 26.31%), although the difference was not statistically significant, and healing time was longer (70.28 vs. 57.68 days, *p* < 0.01). Importantly, biologic treatment was not an independent predictor of bleeding emergency on multivariable analysis. These findings suggest that preoperative inflammatory control may improve local disease behavior, but at the cost of slower wound closure in some patients [22] (Table 1).

## 4. Discussion

The clearest interpretive point is that medical and surgical modalities solve different therapeutic problems. Medical therapy suppresses inflammation, reduces pain and drainage, and may improve quality of life or reduce future lesion formation. Surgery removes existing anatomically diseased tissue and offers the best chance of durable control in a fixed field. A patient with diffuse inflammatory disease but little structural destruction is therefore poorly served by early radical excision, whereas a patient with persistent axillary or inguinal tunnels after multiple antibiotic courses is poorly served by endless medication escalation alone [4,5,7,8,13,14,15,16,17,18,19,23].

In practice, a structural-dominant phenotype is suggested by persistent draining tunnels, fixed fibrotic cords, extensive scarring, recurrent drainage arising from the same anatomical tract, ultrasound-confirmed subclinical tunnel networks, or regionally destructive Hurley III disease. In such patients, repeated escalation of anti-inflammatory therapy alone is unlikely to eliminate established tissue damage, and early referral for deroofing, local excision, or wide excision should be considered. Conversely, patients with predominantly inflammatory nodules and abscesses but limited tunnel burden may derive greater benefit from early systemic escalation.

This distinction also explains why older arguments about whether surgery is “last line” are becoming less convincing. The French recommendation for a medicosurgical approach, the Australasian move away from sequential monotherapy, and the European S2k framing of inflammatory versus predominantly non-inflammatory disease all imply that the best timing of surgery is dictated by lesion biology, not simply by how many medicines have failed [4,5,7].

A related issue is peri-operative biologic use. Historically, some clinicians withheld biologics around surgery because of concerns about infection or wound complications. The HS-specific evidence is now more reassuring for adalimumab. SHARPS found benefit without new peri-operative safety signals, and the neoadjuvant cohort found no independent bleeding effect attributable to biologics, although wound healing was slower. On present evidence, interrupting effective adalimumab solely because surgery is planned appears unnecessary in many patients, provided infection risk, wound plans, and multidisciplinary oversight are appropriate. Evidence of equal strength does not yet exist for every newer biologic, so extrapolation to secukinumab or bimekizumab should remain cautious [20,21,22].

A practical peri-operative approach includes assessment of active infection, smoking status, glycemic control, nutritional status, obesity-related risk factors, and wound-healing capacity. Patients receiving effective biologic therapy for active inflammatory disease may often continue treatment through surgery, particularly when an extensive interruption risks inflammatory rebound. Preoperative ultrasound can assist surgical planning and margin definition. Closure strategy should also be considered carefully because flap and graft reconstruction may have different recurrence and healing profiles than primary closure. Patients should be counseled that available cohort data suggest a potential trade-off between reduced inflammatory recurrence and prolonged wound-healing time when biologics are combined with surgery.

Finally, the surgical literature suggests that “how” surgery is performed matters as much as “whether” surgery is performed. Incision and drainage should remain a rescue maneuver for painful acute abscesses rather than a definitive disease-modifying procedure. Deroofing is effective for recurrent nodules and localized tunnels, often in Hurley I/II or limited Hurley II/III disease. Wide excision is the best-supported option for advanced regional disease, and reconstruction planning influences recurrence. Where available, preoperative ultrasound appears useful for defining margins and may help distinguish true structural recurrence from ongoing inflammatory disease adjacent to the scar [4,7,8,13,15,17,19,23].

A limiting factor for the evaluation of surgical studies are missing widely accepted definitions for recurrence and surgical techniques (Table 2).

## 5. Clinical Recommendations

For mild, localized, predominantly inflammatory HS, topical or intralesional measures, short-course oral antibiotics such as tetracyclines, lifestyle and comorbidity management, and hormonal therapy in selected women remain appropriate initial strategies. Recurrent fixed lesions in otherwise mild disease should prompt consideration of local procedures such as deroofing or limited excision, rather than repeated antibiotic recycling [1,2,3,4,5,6,7,8].

For moderate-to-severe inflammatory HS without a dominant fixed tunnel burden, early biologic-centered therapy is justified. The strongest direct RCT evidence still exists for adalimumab, but secukinumab and bimekizumab now provide robust phase 3 alternatives. Response should be re-evaluated at clinically relevant intervals, and biologic failure should trigger reconsideration of phenotype, adherence, comorbidity, and the presence of occult structural disease rather than simply prolonging ineffective monotherapy [5,7,10,11,12]. Red flags supporting early systemic escalation include diffuse inflammatory involvement across multiple anatomical regions, rapid relapse following antibiotic withdrawal, frequent inflammatory flares, and major quality-of-life impairment disproportionate to visible disease extent.

For persistent tunnels, fibrosis, and scarred regional disease, surgical referral should be early rather than deferred indefinitely. Deroofing or local excision is well suited to discrete recurrent tunnels or nodules. Wide excision remains the best-supported option for extensive localized Hurley III disease or long-standing anatomically destructive disease, especially where chronic structural change dominates the clinical picture. Reconstruction strategy should be planned, not improvised, because recurrence differs by wound closure method [8,13,14,15,16,17,18,19,23]. Practical red flags prompting early surgical referral include recurrent drainage from the same anatomical tract, ultrasound-confirmed tunnel networks, persistent fibrotic cords despite medical therapy, and regionally destructive Hurley III disease.

For advanced mixed disease, a combined pathway is usually preferable: suppress inflammation medically, map the surgical field carefully, proceed to lesion-directed surgery, and continue postoperative inflammatory control. At present, adalimumab has the clearest HS-specific peri-operative evidence base. Multidisciplinary care involving dermatology, surgery, wound care, pain management, and comorbidity screening is likely to give the best patient-centered outcomes [4,5,7,20,21,22].

## 6. Limitations

This review used a structured search strategy but was not conducted as a prospectively registered systematic review, and therefore, its synthesis remains interpretive. The underlying comparative evidence is also intrinsically difficult to integrate: medical trials favor short-term inflammatory endpoints such as HiSCR, whereas surgical studies report local recurrence, wound healing, and anatomical remission over much longer windows. Definitions of recurrence, especially after surgery, are inconsistent across studies, which partly explains the wide variability in reported outcomes [8,13,14,15,16,17,18,19,23]. Future surgical studies should report tunnel recurrence and inflammatory relapse separately, allowing more meaningful comparison between procedures and better differentiation between failure of local surgical control and persistence of underlying systemic disease activity. In addition, much of the surgical literature remains observational and is susceptible to referral bias, center-specific expertise effects, variation in operative technique, differences in disease extent at baseline, and inconsistent definitions of surgical margins. These factors complicate direct comparison between studies and may contribute substantially to variability in reported recurrence rates. The strongest direct comparison between medical and surgical strategies is still relatively sparse. There are no large trials directly randomizing patients to “best medical therapy” versus “best surgical therapy” across the full disease spectrum. Instead, the literature is dominated by medical placebo-controlled RCTs, surgical meta-analyses of observational cohorts, and a smaller number of integrated peri-operative or pragmatic combination studies. Consequently, the present conclusion in favor of combined care is evidence-based, but it still rests partly on triangulation across different study designs rather than on a single definitive head-to-head program [13,20,21,22,23].

Another limitation is temporal: several influential guidelines predate secukinumab and bimekizumab, and even the most recent guidance partly extrapolates from evolving phase 3 evidence rather than from mature long-term comparative trials. Future research should prioritize standardized recurrence definitions, imaging-informed surgical phenotyping, long-term patient-reported outcomes, and truly comparative medicosurgical strategies [1,5,7,11,12,17,23]. Emerging image-recognition artificial intelligence systems have demonstrated strong diagnostic performance across a range of dermatologic conditions and may ultimately contribute to more personalized HS management. Recent studies suggest that multimodal image-analysis systems can approach specialist-level performance for dermatologic image interpretation [23,24]. In HS, future image-recognition systems may help identify visual patterns associated with inflammatory activity, tunnel burden, fibrosis, and scarring that are difficult to standardize during routine examination. Integration of clinical photography, ultrasound findings, disease-severity scores, and longitudinal outcomes may ultimately improve identification of patients most likely to benefit from early biologic escalation, lesion-directed surgery, or combined therapeutic approaches.

## 7. Conclusions

HS should be treated as a disease with two therapeutically distinct but clinically intertwined dimensions: inflammation and structural damage. Medical therapy is indispensable for suppressing inflammatory activity, improving symptoms, and reducing overall disease burden; surgery is indispensable for persistent tunnels, fibrotic tracts, and scarred anatomical fields that are unlikely to resolve with medication alone [4,5,7,10,11,12,13,14,15,16,17,20,21,22]. The best-supported practical model is therefore not medical versus surgical management but timed and phenotype-directed medical plus surgical management. For mild inflammatory disease, medical therapy with selective office-based procedures may be sufficient. For moderate-to-severe inflammatory HS, biologics should be considered early. For localized structurally advanced disease, deroofing or excision should not be unduly delayed. For mixed or severe disease, the best available evidence supports a multidisciplinary medicosurgical pathway, particularly when adalimumab-supported peri-operative integration is feasible [4,5,7,13,14,15,16,17,18,19,20,21,22,23].

## Figures and Tables

**Table 1:** Medical and surgical effect sizes are not directly commensurate because medical trials usually assess short-term inflammatory response, whereas surgical studies assess local recurrence, healing, or anatomical remission over longer periods.

Study	Design and Population	Intervention/Comparator	Main Outcomes	Follow-Up	Effect Size and Interpretation
Kimball et al. 2016 [10]	Two phase 3 RCTs; PIONEER I (*n* = 307) and PIONEER II (*n* = 326); adults with moderate-to-severe HS	Weekly adalimumab vs. placebo	HiSCR at week 12	12 weeks primary	PIONEER I: 41.8% vs. 26.0%; PIONEER II: 58.9% vs. 27.6%. Strongest early phase 3 evidence for inflammatory control
Kimball et al. 2023 [11]	Two identical phase 3 RCTs; SUNSHINE (*n* = 541) and SUNRISE (*n* = 543)	Secukinumab 300 mg q2w or q4w vs. placebo	HiSCR at week 16 and durability to week 52	52 weeks	q2w met primary endpoint in both trials: 45% vs. 34% and 42% vs. 31%; q4w significant in SUNRISE only
Kimball et al. 2024 [12]	Two phase 3 RCTs; adults with moderate-to-severe HS	Bimekizumab pivotal regimens vs. placebo	HiSCR50 at week 16	48 weeks	Week-16 HiSCR50 broadly 48–54% vs. 29–32% placebo across pivotal arms; extends biologic efficacy beyond anti-TNF and IL-17A blockade
Bechara et al. 2021 [20]	Phase 4 peri-operative RCT in moderate-to-severe HS undergoing surgery	Adalimumab plus surgery vs. placebo plus surgery	All-body HiSCR; peri-operative safety	Peri-operative study; week-12 primary	48% vs. 34% all-body HiSCR at week 12; difference 14% (95% CI 0–27%); supports continuing adalimumab around surgery
Aarts et al. 2023 [21]	Pragmatic real-world RCT; 31 patients per arm	Adalimumab plus surgery vs. adalimumab alone	IHS4 and DLQI change	12 months	IHS4 change −19.1 vs. −7.8 (*p* < 0.001); DLQI change −8.2 vs. −4.0 (*p* = 0.02); combination outperformed monotherapy
Salvador-Rodríguez et al. 2020 [22]	Prospective cohort; surgery plus biologic (*n* = 21) vs. surgery alone (*n* = 38)	Excision with secondary intention, with or without neoadjuvant biologic	Recurrence, healing time, complications	24 weeks	Recurrence 9.5% vs. 26.3% (NS); healing 70.3 vs. 57.7 days (*p* < 0.01); biologic cohort healed more slowly but trended to less recurrence
Riddle et al. 2021 [13]	Systematic review (59 studies) and meta-analysis (33 studies)	Surgical approaches compared across studies	Recurrence after surgery	≥1 year for pooled recurrence analyses	Wide excision pooled recurrence 8% (95% CI 2–16%); local excision 34%; flap repair had the lowest pooled recurrence within wide/radical excision
Cucu et al. 2025 [16]	Systematic review/network meta-analysis after wide excision	Closure strategy comparisons	Recurrence by closure method	Variable	Compared with primary closure, local/distant flap OR 0.450 and split-thickness graft OR 0.549 for recurrence; supports planned reconstruction in selected cases
Deckers et al. 2018 [17]	Retrospective single-center study; 84 patients, 253 procedures	Wide excision with secondary intention healing	Recurrence and remission by anatomical area	Mean 36.2 months	Recurrence in 37.6% of procedures; total remission in 49%; meaningful local control but not universal cure
García-Moronta et al. 2026 [19]	Retrospective single-center cohort; 165 patients, 206 procedures	Predominantly wide excision with secondary intention healing	Recurrence phenotype, healing time, predictors	2018–2024 cohort follow-up	Overall recurrence 18.5%; tunnel recurrence 8.3%; excised area independently predicted recurrence (OR per cm^2^ 1.03)

**Table 2:** Recommendations are summarized from the original guidelines and the comparative review in Refs. [1,2,3,4,5,6,7,8]. Later guidelines increasingly shift from a strict therapeutic ladder to lesion- and phenotype-directed multimodal care.

Guideline	Medical Pathway	Procedural/Surgical Pathway	Comparative Takeaway
British Association of Dermatologists 2018 [1]	Topicals for mild disease; tetracyclines generally for 12 weeks; adalimumab after inadequate conventional systemic therapy	Incision and drainage for symptom relief; wide local excision for severe localized disease; deroofing is less explicitly emphasized than in later guidelines	More stepwise and conservative; reflects the pre-secukinumab and pre-bimekizumab era
North American Part I/II 2019 [2,3]	Topical/intralesional therapy, tetracyclines, clindamycin-rifampicin, hormonal options in selected women, adalimumab as first-line biologic, intravenous ertapenem as rescue/bridge	I&D only for acute abscesses; deroofing for recurrent nodules/tunnels; wide local excision for advanced localized disease	Strong procedural integration; one of the clearest early models of parallel medical and surgical care
French Society of Dermatology 2021 [4]	Stage-based antibiotics; anti-TNF may be proposed for Hurley II/III with reassessment at 3–6 months	Surgery is considered locally curative for specific lesions and recommended as part of care rather than as an afterthought	Explicitly “medicosurgical”; severe disease should receive specialized multidisciplinary care
Australasian guideline 2025 [5]	Discourages sequential monotherapy; emphasizes multimodal escalation; includes OCP/spironolactone in selected patients; adalimumab, secukinumab, and bimekizumab included for moderate/severe disease	Laser hair reduction, local excision/deroofing early in the pathway; IV ertapenem can bridge to wide surgery	Strongly modern, phenotype-led and multimodal; highly relevant to Australasian practice
European S2k Part 2 2024 [7]	Distinguishes inflammatory disease managed primarily with medication; highlights tetracyclines as an alternative to clindamycin-rifampicin; recognizes adalimumab, secukinumab, and bimekizumab	Predominantly non-inflammatory structural disease is directed towards surgery according to the Hurley stage and anatomical site	Probably the clearest formal articulation of inflammatory versus structural disease phenotypes
Comparative review of international guidelines 2021 [8]	Consensus across guidelines: topicals for mild disease, systemic antibiotics for wider or more active disease, biologics for refractory moderate/severe disease	Deroofing and wide local excision repeatedly emerge as the most consistently supported procedures	Helps explain where guidelines converge despite regional variation

## Data Availability

No new data were created or analyzed in this study.
